# Translational Stroke Research Review: Using the Mouse to Model Human Futile Recanalization and Reperfusion Injury in Ischemic Brain Tissue

**DOI:** 10.3390/cells10123308

**Published:** 2021-11-25

**Authors:** Emilia Conti, Benedetta Piccardi, Alessandro Sodero, Laura Tudisco, Ivano Lombardo, Enrico Fainardi, Patrizia Nencini, Cristina Sarti, Anna Letizia Allegra Mascaro, Marzia Baldereschi

**Affiliations:** 1 Neuroscience Institute, National Research Council, Via G. Moruzzi 1, 56124 Pisa, Italy; conti@lens.unifi.it (E.C.); allegra@lens.unifi.it (A.L.A.M.); 2 European Laboratory for Non-Linear Spectroscopy, Via Nello Carrara 1, 50019 Sesto Fiorentino, Italy; 3 Neurofarba Department, University of Florence, Via G. Pieraccini 6, 50139 Florence, Italy; alessandro.sodero@unifi.it (A.S.); lauratudisco89@gmail.com (L.T.); cristina.sarti@unifi.it (C.S.); 4 Department of Biomedical, Experimental and Clinical Sciences, University of Florence, Viale Morgagni 50, 50134 Florence, Italy; ivano.lombardo@gmail.com (I.L.); enrico.fainardi@unifi.it (E.F.); 5 Stroke Unit, Careggi University Hospital, Largo Brambilla 3, 50134 Florence, Italy; nencinip@aou-careggi.toscana.it; 6 Neuroscience Institute, National Research Council, Via Madonna del Piano 10, 50019 Sesto Fiorentino, Italy; marzia.baldereschi@in.cnr.it

**Keywords:** brain, ischemic stroke, reperfusion, futile recanalization, mechanical thrombectomy, translational stroke research

## Abstract

The approach to reperfusion therapies in stroke patients is rapidly evolving, but there is still no explanation why a substantial proportion of patients have a poor clinical prognosis despite successful flow restoration. This issue of futile recanalization is explained here by three clinical cases, which, despite complete recanalization, have very different outcomes. Preclinical research is particularly suited to characterize the highly dynamic changes in acute ischemic stroke and identify potential treatment targets useful for clinical translation. This review surveys the efforts taken so far to achieve mouse models capable of investigating the neurovascular underpinnings of futile recanalization. We highlight the translational potential of targeting tissue reperfusion in fully recanalized mouse models and of investigating the underlying pathophysiological mechanisms from subcellular to tissue scale. We suggest that stroke preclinical research should increasingly drive forward a continuous and circular dialogue with clinical research. When the preclinical and the clinical stroke research are consistent, translational success will follow.

## 1. Introduction

Intravenous thrombolysis (IVT) and mechanical thrombectomy (MT) are established treatments proven to reduce disability after acute ischemic stroke by salvaging the brain. Even in the case of complete vessel recanalization, some patients remain functional dependent, which is thus called “futile recanalization” (FR) [1,2,3]. Moreover, some patients develop early complications, among which hemorrhagic transformation (HT) and cerebral edema (CE) are the most feared.

HT encompasses a broad spectrum of severity grades ranging from small areas of petechial hemorrhage to massive space-occupying hematomas. From the clinical point of view, HT has been divided into symptomatic and asymptomatic, a distinction that is important while evaluating the overall risk-to-benefit ratio of revascularization treatments.

CE is a severe complication of acute ischemic stroke and is the cause of death in 5% of all patients with cerebral infarction [4,5]. Edema causes tissue shifts and increased intracranial pressure that can cause death, usually between the second and the fifth day after stroke onset [6,7]. A large and potentially life-threatening infarct in the territory of the middle cerebral artery is often called a malignant middle cerebral artery infarct [4]. If not treated with reperfusion therapies, ≈50% to 80% of patients with this condition die, despite basic life support strategies. Surgical treatment by early decompressive hemicraniectomy decreases mortality, and decompressive hemicraniectomy is recommended by leading practice guidelines in selected patients [8]. Swelling and infarct growth each contribute to total stroke lesion growth in the days after stroke and should be considered a predictor of poor outcome even in patients with moderately sized stroke [9].

A critical role in the molecular mechanisms determining HT and CE is the disruption of the neurovascular unit (NVU), a dynamic terminal structure configuring an elaborate vascular network. The NVU is composed of an arteriole and its endothelial cells, basal lamina matrix, astrocyte end-feet, pericytes, astrocytes, neurons and their axons, and supporting cells (microglia and oligodendroglia) [10,11], and allows neurons to regulate micro vessels to support the metabolic needs of the tissue. The specialization and cellular composition of the NVU varies spatially along the arteriole–capillary–venule axis in order to allow local neurovascular coupling [12]. Indeed, more recently, a new concept of NVU has been proposed, identifying this complex interaction of segmentally diverse functional modules aimed to coordinate the entire brain vascular system, reacting to central and peripheral signals to maintain homeostasis of the brain, in health and disease [13].

During the ischemic insult, NVU participates in the reperfusion battleground occurring between the ischemic core and the surrounding salvageable tissue. Endothelial basal lamina dissolution starts as soon as 2 h after the onset of ischemia and is rapidly followed by an increase in Blood–Brain Barrier (BBB) permeability [14]. The early phase of BBB leakage occurs at 6 h from symptom onset, while there is a delayed secondary opening that occurs during the neuroinflammatory response (24–72 h after the ischemic insult). The prevailing view attributes the biphasic increase in BBB permeability to the disintegration and redistribution of tight junctions (TJs). However, recent studies suggest that increased endothelial transcytosis precedes and is independent of TJs disintegration [15]. BBB disruption following ischemic stroke contributes to HT, CE, secondary injury, and mortality. Clinical studies show no apparent increase in the risk of CE in ischemic stroke patients receiving IVT. However, there is experimental evidence that IVT could impair the BBB and contribute to reperfusion injury [16].

Age, stroke severity [17,18], and procedure delay [19], are the main predictors of FR.

Notably, the problem of selection for reperfusion therapies also persists after the introduction of multiparametric imaging techniques, such as multimodal Computed Tomography (CT) protocol and magnetic resonance (MR) imaging. These widely used techniques include: 1—non-contrast CT (NCCT) to detect intracerebral hemorrhage and early ischemic changes, quantified by a semiquantitative method known as ASPECTS (Alberta Stroke Program Early CT Score); 2—CT Angiography (CTA) to identify the occlusion site and to assess collateral circulation with a single-phase (sCTA) or, better, multiphase (mCTA) techniques; 3—CT perfusion (CTP) to define the size of infarct core and ischemic penumbra and consequently their mismatch using its capability to discriminate the different functional components of the ischemic area [20,21,22,23,24]. Likewise, MR findings can improve the ability to select patients for novel treatment options properly by using experimental approaches measuring selected biochemical parameters in the brain, in addition to the most common sequences (i.e., perfusion and diffusion) [25,26]. Nonetheless, MR application in the clinical field, despite being feasible, is constrained by the limited availability of this exam, which is hard to reconcile in the context of a time-dependent disease [27]. Aside from clinical and radiological parameters, blood biomarkers may also serve as a practical tool to represent the pathophysiology status before clinical deterioration. A recent observational study showed that the increased levels of some circulating biomarkers (particularly metalloproteinases and inflammatory biomarkers, such as C-Reactive Protein) were independent predictors of FR in acute ischemic stroke patients after recanalization by endovascular treatments [28]. Precision medicine, the initiative to replace a one-size-fits-all approach designed for the average patient with treatments tailored to account for unique differences among individuals, shifts paradigms across many fields of medicine. The treatment of ischemic stroke due to large vessel occlusion (LVO) offers a compelling illustration. Individual intrinsic differences in the flow capacity of collateral vessels, degree of chronic ischemic disease, ischemic preconditioning, oxidative stress tolerance, microvascular blood flow regulation, and other factors influence each patient’s response to treatment [29]. Preclinical research may help understand the neuronal and vascular underpinnings of FR, identify potential treatment targets, and lead to clinical translation.

To better explain the aim of this review, we present three real-world LVO stroke patients with similar risk profiles and fully reperfused by combined IVT and MT, as paradigmatic examples capable of stimulating stroke clinician’s specific questions to preclinical research. All patients’ data and imaging were obtained under informed consent.

## 2. From Bedside

**Figure 1:**
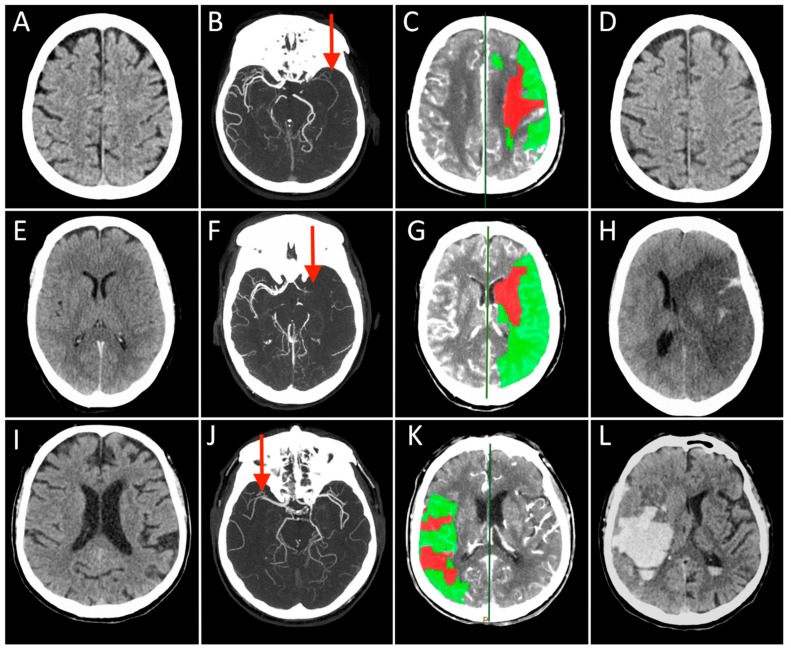
Each row represents a single patient’s images derived in different modalities and timepoints (Case 1 presented in (**A**–**D**), Case 2 in (**E**–**H**), and Case 3 in (**I**–**L**)). The first three columns on the left show the Non-Contrast-CT (NCCT), multiphase CT Angiography (CTA), and CT Perfusion (CTP) performed at hospital arrival, while the last column on the right displays the NCCT acquired at 24 h after stroke. On presenting NCCT (**A**,**E**,**I**) no early ischemic changes can be seen in the brain tissue in Case 2 and 3 (ASPECT score = 10), while a tissue swelling was detected in the right insular lobe in Case 1 (ASPECT score = 9, not shown). The mCTA images identified proximal (M1 segment) Middle Cerebral Artery (MCA) occlusion in the left hemisphere in (**B**,**F**), and contralateral side in (**J**) (red arrows). CTP showed in all cases a small infarct core corresponding to CBV lesion (red) and a large ischemic penumbra consisting of the difference between MTT and CBV lesions (green), representing the expected “salvageable” tissue after recanalization. In these patients, CBV lesion volume ≤ 50% of MTT lesion size. All patients were treated with combined IVT and MT, obtaining a complete recanalization, but the 24 h NCCT showed three different conditions: no ischemic lesion visible (**D**), complete MCA territory infarct associated with a massive cerebral oedema (**H**), and a vast hemorrhagic transformation of the ischemic lesion.

Case 1: A 73-year-old man with a history of hypertension, dyslipidemia, and prior myocardial infarction presented to the emergency department after the sudden onset of right-sided weakness and difficulty speaking. NCCT showed early ischemic changes in the left insular territory with an ASPECT score of 9 (Figure 1A). The mCTA demonstrated occlusion of the distal left M1 segment of the middle cerebral artery (MCA) with good collateral circulation (Figure 1B). A large penumbra in the left MCA territory with CBV lesion volume ≤ 50% of MTT lesion size (Figure 1C). After IVT, started 115 min after symptom onset, he underwent MT with complete recanalization (onset to recanalization time 280 min). In the 24 h follow-up CT scan, no ischemic lesion was visible (Figure 1D), and the patient experienced a great clinical improvement over the subsequent 3 days. At the 3-months follow-up, the patient was functionally independent.

Case 2: A 71-year-old woman presented to a local emergency department 1 h after the sudden onset of speech difficulties and hemiparesis involving the right face, arm, and leg. She had a history of poorly controlled cardiovascular risk factors. NCCT showed a left MCA hyperdense sign (Figure 1E). The mCTA demonstrated a proximal left MCA occlusion (Figure 1F). CTP revealed a large penumbra in the left MCA territory with CBV lesion volume ≤ 50% of MTT lesion size (Figure 1G). She had no contraindications for IVT that was started 160 min after symptom onset. The patient then underwent endovascular treatment with complete recanalization that was not followed by clinical recovery (onset to recanalization time 300 min). Serial NCCT scans demonstrated progressive edema with mass effect in the left MCA distribution and 12 mm of midline shift (Figure 1H). She required endotracheal intubation and was admitted to the neurocritical care unit. The patient died the next day.

Case 3: A 70-year-old man had a sudden onset of left hemiplegia and forced eye deviation to the right. His medical comorbidities included hypertension, dyslipidemia, type 2 diabetes mellitus, and coronary artery disease. On neurological examination, he was awake, unable to communicate or follow commands. He had forced right gaze deviation, left hemianopsia, moderate left arm and leg weakness. NCCT identified hyperdense right MCA sign. The ASPECT score was 10 (Figure 1I). The mCTA showed an occlusion of the right proximal M2 segment of the MCA with a good collateral flow (Figure 1J). CTP demonstrated a large penumbra in the right MCA territory with CBV lesion volume ≤ 50% of MTT lesion size (Figure 1K). He received IVT after 90 min from clinical onset and underwent MT with complete recanalization (onset to recanalization time 215 min). The patient showed initial mild clinical improvement that was followed by both a rapid deterioration of consciousness and worsening neurological conditions with extensor posturing. Follow-up NCCT demonstrated an evolving infarct of the right MCA territory with hemorrhagic transformation and 8-mm midline shift (Figure 1L). He underwent surgical hematoma drainage with partial improvement. The patient was unable to walk and dependent on daily activities at the 3-month follow-up.

The three cases presented here show rather different clinical–functional outcomes despite similar neurological severity, neuroimaging at onset, and recanalization degree. Different treatment delays and clinical parameters might only partially account for differences in outcomes. The potential causes underlying this phenomenon are probably multifactorial but poorly understood. Ischemic changes at the tissue level appear to play a critical role and include reperfusion injury and ischemia-related microcirculatory dysfunction. It is of paramount importance to understand in depth what is there inside the hypoperfused tissue in order to find new predictors of clinical deterioration/FR and, consequently, specific prevention strategies.

## 3. From Bench

In the past few decades, the development of animal stroke models helped dissect the mechanism of neurovascular disruption as the main factor in determining the clinical outcome.

Interestingly, rodent stroke models and study designs revealed numerous potential targets for novel stroke therapies; yet subsequent clinical stroke trials failed to confirm promising preclinical findings. In the past, the National Institute of Neurological Disorders and Stroke (NINDS) tried to address these gaps, searching for new “vision and opportunities” in translational stroke research [30]. In line with their guidelines, a continuous comparison between clinical and preclinical research is necessary in order to be able to answer specific clinical questions.

With reference to the clinical cases presented, we aim to investigate to what extent preclinical research can answer the following questions:

(1) Are there experimental animal models capable of reproducing human LVO stroke and subsequent recanalization? Are there animal models for age, sex, and comorbid human conditions?
(2) Can animal stroke models assess early endpoints, such as hemorrhagic transformation or cerebral edema?
(3) Which mechanistic insight could be given by preclinical research to explain the different evolution of hypoperfused tissue?

In order to answer these questions, we have reviewed (A) the main preclinical models of stroke, highlighting both the similarities with human LVO stroke and subsequent recanalization, and possible limitations on the use of animal models; (B) how preclinical research assesses the most frequent clinical complication (i.e., HT and CE); (C) how novel optical imaging techniques can provide high resolution structural and functional investigations thus help explain reperfusion consequences.

### 3.1. Experimental Animal Model of Occlusion/Reperfusion: Pros and Cons

Experimental stroke models are widely used to investigate the events associated with both the cellular response within the ischemic or healthy cortical area and the mechanisms of recovery in the peri-infarct regions. In particular, animal models of focal cerebral ischemia allow identifying the critical cerebral blood flow thresholds responsible for cell death, electrical failure, inhibition of protein synthesis, energy depletion, and thereby the lifespan of the potentially salvageable penumbra [31].

Very common rodent models of ischemic stroke include the middle cerebral artery occlusion (MCAO), endothelin, and photothrombosis. Each of those partially recapitulates substantial features of human LVO stroke and recanalization, as described below.

**Figure 2:**
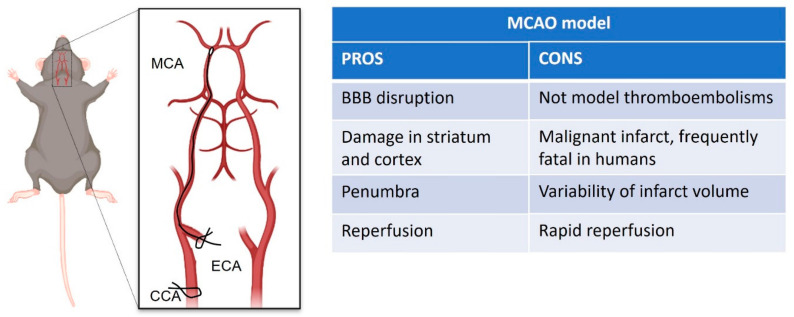
Schematic representation of intraluminal suture of middle cerebral artery occlusion (MCAO) stroke model, created with Biorender.com (accessed on 1 September 2021). On the right pros and cons table of the model.

The intraluminal suture of the middle cerebral artery (MCA, Figure 2) developed by Koizumi et al. [32] and later modified by Longa and colleagues [33] has become a widespread model of choice for mimicking middle cerebral artery occlusion (MCAO) in rodents, due to the minimally invasive technique involved, and ability to allow reperfusion post-occlusion [34]. This model is able to reproduce ischemic stroke and subsequent neuronal cell death, cerebral inflammation, and BBB disruption [35,36]. The damage that results from the interruption of blood flow is mainly in the striatum and cortex [33]. Such extensive damage is akin to a malignant infarct in humans, which is frequently fatal despite treatments [37]. Since this technique produces a considerable volume of potentially salvageable penumbra (area with partial vascular flow) [38,39,40], it is a useful model for investigating the impact of therapeutic approaches on either the volume or lifespan of the penumbra or tissue salvage following the reperfusion [41,42]. Indeed, early restoration of blood supply is a major determinant of the severity of ischemic injury in humans [43] and can result in the success of thrombolytic therapy following acute ischemic stroke in some individuals [44]. However, the retraction of the suture in preclinical research promotes prompt reperfusion of the MCA, whereas in human ischemic stroke, reperfusion typically occurs gradually [45]. The prompt reperfusion induced in this model better approximates what occurs in endovascular thrombectomy [46]. On the other hand, as highlighted by clinicians, reperfusion can trigger deleterious biochemical processes that may antagonize the beneficial effects of blood flow restoration [47,48]. In particular, the recent study of Xu and collaborators [49] that compares rapid versus gradual recanalization, highlighted that flow-controlled reperfusion could be a cerebro-protective strategy after focal cerebral infarction, alleviating cerebral ischemia reperfusion injury, with the evidence of significantly reduced neurological deficit, histopathological damage, infarct size, and neuronal apoptosis in MCAO rats.

Though this model highly reproduces the reperfusion by endovascular thrombectomy, it has several drawbacks, such as low reproducibility due to the variability of the infarct volume depending on the size of the sutures. Another drawback of this model is that considering the nature of blood flow interruption, it is not suitable to mimic thromboembolism [36].

**Figure 3:**
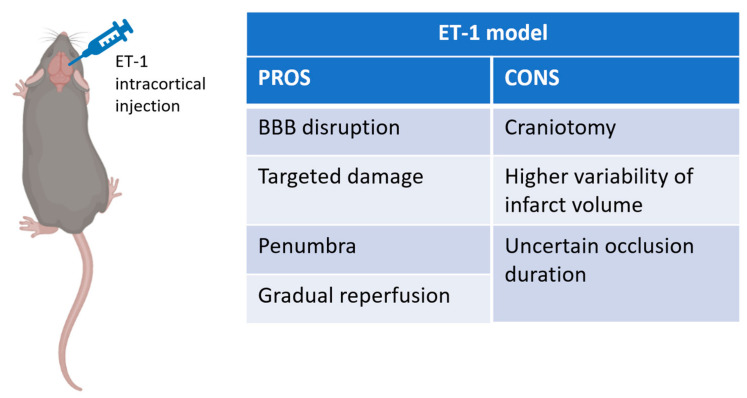
Schematic representation of Endothelin-1 stroke model, created with Biorender.com (accessed on 1 September 2021). On the right pros and cons table of the model.

The endothelin-1 model of focal stroke (Figure 3) is based on the local application of exogenous endothelin-1 (ET-1), a potent and long-active vasoconstrictive peptide, which induces stroke and cell death after sustained vasoconstriction with reperfusion. The main advantages of this model include the ability to perform the procedure quickly, to control artery constriction by modulating the dose of ET-1 delivered, avoiding manipulation of the extracranial vessels supplying blood to the brain, as well as gradual reperfusion rates that more closely mimic the spontaneous and thrombolytic reperfusion in humans [50,51,52]. On the other hand, the ET-1 model has disadvantages that include the need for a craniotomy, as well as higher variability in stroke volume [53]. Another important consideration is that although reperfusion is a common occurrence in human stroke, the duration of occlusion for ET-1 induced MCAO may not closely mimic that of the human stroke where many patients have partial reperfusion over a period of hours to days following occlusion [54,55]. Finally, it is unclear how much penumbral tissue this model produces.

**Figure 4:**
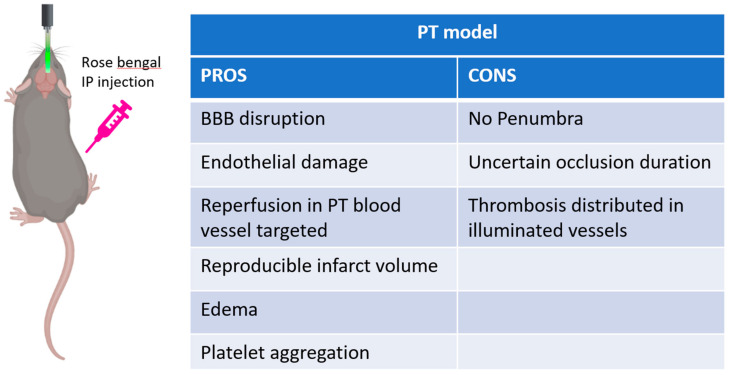
Schematic representation of photothrombotic stroke model, created with Biorender.com (accessed on 1 September 2021). On the right pros and cons table of the model.

In the photothrombotic model (Figure 4), a photosensitive dye, usually Rose bengal, is injected systemically. A light source can be applied on the intact skull with no need for craniotomy, which allows targeting any area of interest within the dorsal cortex in a reproducible and non-invasive way. As a consequence of illumination, the dye is activated, producing singlet oxygen; these oxygen intermediates induce endothelial cell membrane peroxidation, leading to platelet adhesion and aggregation, and eventually to the formation of thrombi which determine local cerebral flow interruption [56]. This procedure generates a sizable volume of penumbra when assessed acutely with MRI [57]. Depending on the procedure applied, the target of photothrombosis can be a single blood vessel or a more extended region of the cortex. Indeed, photothrombosis is a non-canonical ischemic model that is capable of inducing lesions in more superficial vessels. In spite of this, the photothrombotic damage shares essential mechanisms occurring in human stroke. Similar to artery occlusion in human stroke, platelet aggregation and clot formation determine interruption of blood flow in the irradiated area [56]. Likewise, this model also shares essential inflammatory responses as in MCAO [58]. Another important aspect of this technique is that it allows the reperfusion of the occluded blood vessel [59,60]. Though this alternative strategy is not common, a previous study has shown that illumination with a low-energy-density ultraviolet laser is capable of inducing a vascular dilation of the occluded blood vessel. These mechanisms facilitate the formation of microscopic multiple, progressively enlarging channels in the thrombus that lead to recanalization of platelet-occluded arteries [59,61]. This procedure induces a gradual increment of the blood flow in the region of the brain downstream the targeted blood vessel. Indeed, compared to the MCAO technique in which the removal of the intraluminal suture induces a sudden restoration of blood perfusion [61], the disintegration of the obstruction with light induces progressive reperfusion of the region downstream the occluded blood vessel. This aspect represents a key value of this technique since it better approximates the effect of thrombolytic therapies applied to humans. To conclude, the non-mechanical approach of this model has the fundamental advantage of maintaining the dura mater intact, preserving the intracranial pressure.

#### Limitations on Animal Models Use for Age, Sex, and Comorbid Human Conditions

Though many efforts have been made to develop animal stroke models resembling human ischemia [30], biological variables such as age, sex, and comorbidities that profoundly affect the clinical outcome in patients with ischemic stroke are very hard to reproduce. Unlike the patient population usually enrolled for clinical trials, preclinical studies are conducted in highly uniform groups of animals with a homogeneous genetic background. Indeed, the more common use of healthy young-adult rodents allows the scientific community to investigate basic shared pathophysiological mechanisms without possible confounding effects of aging. Particularly in aged animals, long-term surviving studies represent a hard challenge since the high mortality of the sample does not always allow data to be collected at all time points. Indeed, since the mortality rate is related to the infarct extension, the surviving animals are representative of a subgroup of the entire cohort, the one with a moderate infarct size. Another important aspect that should be considered in translational studies is that the aged brain not only responds to an insult or injury differently but also exhibits less restorative capacity in comparison to the healthy and young brain. Additionally, sex can influence both clinical consequences of ischemia and clinical approaches that can be applied. However, the use of females before menopause can be affected by the estrogen’s interference, while the use of aged females after the cessation of the estrous cycle represents only a more selective model for a restricted population. Another challenge for preclinical trials regards the capability to build stroke animal models, including comorbidities known to be possible contributing causes by clinicians. Indeed, stroke occurs due to a variety of vascular pathologies and injury mechanisms, some of which are difficult to model in animals. In this framework, several models of hypertension have been developed to investigate hypertensive cerebral damage, although these models present severe disadvantages since the hypertension induced is limited in time (usually weeks) and does not mimic the long-lasting impact on the brain of the human disease [62]. Moreover, due to the age and/or the presence of comorbidities, the time required to perform experiments dramatically increases [63]. In detail, the great effort of preclinical research to reproduce pathological models of hyperlipidemia, obesity, diabetes, just to cite a few of the most common comorbidities, requires that rodents have to be maintained on a particular diet for several weeks/months. Moreover, precision surgeries, such as intraluminal MCAO and embolic stroke, in aged rodents become methodologically challenging due to the physiological alterations of cerebral blood vessels [63]. The variation of intraluminal blood vessel diameter requires modification of occluding filament caliber according to animal weight [64]. Another technical complication related to the use of aged animals consists in maintaining a constant level of anesthesia during the surgery [65,66,67]. Indeed, the use of common anesthetics, such as isoflurane and barbiturates, can both interfere with several signaling pathways related to post-stroke treatment [68,69] and increment the physiological cerebral blood flow [70]. In a recent study, Balbi and colleagues validated the photothrombotic occlusion through a permanent transcranial window in awake mice as a reliable stroke model free from anesthesia confounding factors [71].

Finally, several complications can occur after the surgery requiring careful and more frequent post-surgery monitoring [63] and increasing the mortality of aged and comorbid animals [72]. Some expedients can increase the success of these procedures. On the one hand, the reduction in the occlusion time results in reduced mortality in the intraluminal stroke model of aged and comorbid animals. On the other hand, different types of strategies could be applied to induce an ischemic stroke. In particular, the photothrombotic stroke model shows a reduced mortality rate compared to MCAO. For these reasons, up to now, the combination of ischemia-related pathologies with animal stroke models still presents substantial troubles. Given all of this, despite the aforementioned complications, translational research would certainly benefit from implementing ischemia animal models with biological variables. Although it has already been ascertained that age, sex, and comorbidities are important factors to consider when establishing the efficacy profile of individual intervention, up to now, most preclinical studies fail to evaluate their contribution, the main reasons being the high costs to acquire and maintain aged and/or comorbid animals [63], in addition to the increased mortality of these mice after stroke. As a consequence, the vast number of biological variables (i.e., age, sex, and potential comorbidities and multimorbidity combinations) that characterize stroke patients and their combinations are hard to be represented in experimental settings [73].

Nevertheless, preclinical research makes great efforts to develop animal models that better resemble the multifaceted scenario of stroke patients by exploiting aging animals [74,75,76], genetically modified animal lines [77,78,79,80], and novel fine-tuned experimental protocols to overcome the complications of some procedures [64,71,79].

### 3.2. Hemorrhage and Edema Evaluation in Preclinical Research: From the Macro- to Micro-Scale

#### 3.2.1. Ex-Vivo Studies

After cerebral vascular occlusion, many events occur as a direct consequence of the blood flow reduction, such as energy failure, excitotoxicity, increase in intracellular calcium levels, and generation of free radicals. Taken together, all these are potential causes of BBB disruption, which leads to vasogenic CE, inflammation, and possibly HT after stroke [81]. While in clinics, HT and CE play crucial roles in determining patients’ prognosis, in animal studies, the assessment of stroke severity is carried out through behavioral experiments combined with lesion volume evaluation.

Nevertheless, many Ex Vivo and In Vivo experimental methods have been proposed for assessing the disruption of the BBB in animal models [82,83]. More in detail, literature in this field ranges from whole-brain approaches, such as magnetic resonance [84,85,86], to single blood vessel investigation through in vivo fluorescence imaging [82,87].

**Figure 5:**
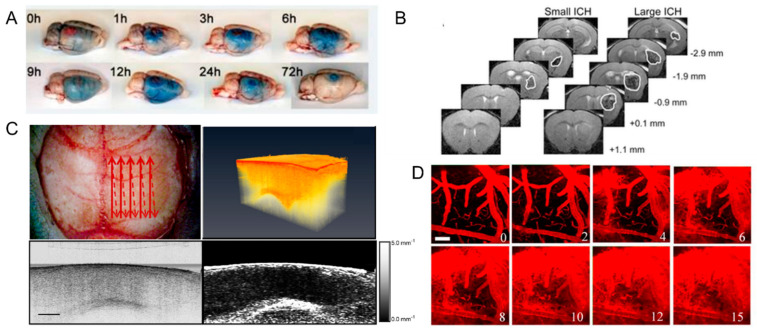
Hemorrhage and edema evaluation in preclinical research: (**A**) Evaluation of Evans Blue extravasation at different time points from the induction of photothrombosis. Modified from Stoll et al., 2008. (**B**) Representative images of T2-MRI scans (+1.1 mm to −2.9 mm relative to bregma) in a mouse with small hematoma and a mouse with large hematoma at 6 h after induction of intracerebral hemorrhage. The boundary between hematoma and surrounding tissues is indicated by a solid white line. Modified from Matsushita et al., 2013. (**C**) Upper left: red arrows depict the optical beam scan pattern for three-dimensional 3-D imaging of the sample; upper right: 3-D volume of in vivo mouse brain rendered from OCT volumetric scan; lower left: sagittal OCT intensity image of in vivo mouse brain and corresponding (lower right) attenuation image. Scale bar = 0.5 mm. Modified from Rodriguez et al., 2014. (**D**) Depth projection images illustrate the transient BBB disruption induced by microbubbles and focused ultrasound at 0.6 MPa (scale bar: 100 μm). Modified from Nhan et al. 2013.

In many studies, hemorrhage assessment is performed through ex vivo evaluation exploiting staining that differentiates between metabolically active and inactive tissue [88] or the diffusion of injected dye within the brain tissue [83,89,90]. The first method, employed by Zhao and collaborators [88], allows the evaluation of hemorrhage 24 h after the occlusion of MCA by staining brain slices with a redox indicator, Triphenyl tetrazolium chloride (TTC). More in detail, they evaluated the presence of hemorrhagic tissue in the unstained slices, and then they compared the hemorrhagic tissue to the area of infarction observed after the staining with TTC. They then quantified the hemorrhagic tissue through a commonly used image analysis system. The second method also applied in vivo [91], employed the Evans Blue (EB) dye to estimate the extravasation. Since intravenously injected EB binds serum albumin in vivo, the presence of blue-stained cerebral tissue, assessed through an ex vivo evaluation, reveals the loss of BBB integrity and the extension of extravasation (Figure 5A). The extension of BBB breakdown is estimated based on the detection [84] or quantification [83,89,90] of EB diffusion into the brain tissue. To evaluate the extension of EB staining, Stoll and collaborators [84] performed a qualitative morphologic examination of fixed brain tissue after the induction of photothrombotic lesion in rats at different time points. Then Park and colleagues characterized in a rat model of subarachnoid hemorrhage the BBB permeability by applying spectrofluorophotometry [83] and the brain edema by measuring brain water content 24 h after the injury. Though these protocols offer a wide range of alternatives to evaluate the extravasation of EB, since all these procedures are performed ex vivo, they do not allow evaluation at different time points on the same animal.

#### 3.2.2. In-Vivo Studies

To overcome this significant limitation, different imaging techniques are applied to perform in vivo evaluations of post-stroke hemorrhage and edema with different resolutions and scales. Among all, MRI technology represents a methodological link between clinical and preclinical research. The main advantage of high-resolution preclinical MRI is that it provides a direct display of morphological changes in cerebral architecture linked to HT and CE with high precision and non-invasiveness in the entire brain. Xiong and collaborators investigate cortical inflammatory edema in a rat model of stroke [86], exploiting the capability of MRI to dynamically obtain detailed pathological information of neuronal injuries and microglial reaction in vivo. Another study [92] described the progression over time of brain injury exploiting the capability of MRI to extract quantitative and qualitative parameters to assess alteration of cerebral blood flow and BBB permeability. More recently, Matsushita and colleagues [93] demonstrated, through an MRI-based analysis, a tight relationship between intracerebral hemorrhage and stroke clinical development conferring to MRI investigations the capability to predict neurological dysfunction and animal mortality (Figure 5B). Despite the great advantages provided by MRI, this technique is not extensively used in animal studies since the equipment is really expensive. Alternatively, a more reasonably-priced approach for detecting CE in vivo exploits optical coherence tomography (OCT) [94,95,96]. Based on the fact that the optical scattering of tissue is influenced by the composition of the tissue itself, it will change accordingly with the increase in water content during cerebral edema. OCT allows for the cross-sectional acquisition of biological tissue with high resolution (micrometer) and tissue penetration of the order of millimeters [94,95]. Rodriguez and collaborators [94] demonstrated the possibility of employing OCT to detect optical changes correlated with cerebral edema in an in vivo water intoxication model (Figure 5C). This study revealed an advancing alteration of the cerebral cortex attenuation coefficient that goes hand in hand with the edema progression. Moreover, they used Doppler OCT imaging to detect a decrease in cerebral blood flow due to blood vessel compression during severe brain swelling [94]. Moreover, different from clinical research, preclinical investigations allow the observation of BBB disruption with high resolution by exploiting fluorescence imaging techniques in vivo. In particular, two-photon fluorescence microscopy (2PFM) combined with fluorescent staining of the vasculature provides a longitudinal evaluation on the blood vessels’ permeability within the mouse brain cortex through a cranial window. Moreover, this approach offers sufficient temporal and spatial resolution to track transient changes in BBB permeability at a microvascular level. Proof-of-concept in monitoring BBB disruption using 2PFM has been demonstrated by Raymond et al. [97,98]. In that study, the authors injected fluorescent dyes (e.g., Texas Red, Oregon Green) for the visualization of the microvasculature and transmitted ultrasound from the ventral surface of the brain to induce BBB alterations. These studies characterized the microscopic leakage patterns qualitatively but did not attempt to quantify the rate of agent delivery. Then by extracting and correlating intravascular and extravascular signals from the time-lapse 2PFM images, Nhan and collaborators [87] (Figure 5D) demonstrated a quantitative approach to analyze the 2PFM images after BBB disruption. In detail, they characterized the apparent permeability by comparing the intra- and extravascular fluorescence between two time intervals after the injection of a tracer. Recently, Allegra Mascaro et al., 2019 [82] applied this protocol in order to investigate BBB permeability in a mouse model of photothrombotic stroke in the primary motor cortex. More in detail, they investigated the extravasation of a low molecular weight dye (3KDa Texas red dextran) at two different time points (15 and 30 days after the injury) after stroke. Though 2PFM does not allow whole organ investigation, the great advantage of this approach is the capability to perform longitudinal studies, thus offering the possibility to monitor the integrity of the BBB with high precision even in the chronic phase after stroke.

### 3.3. Optical Imaging to Investigate Structural and Functional Plasticity in Mouse Models of Stroke

**Figure 6:**
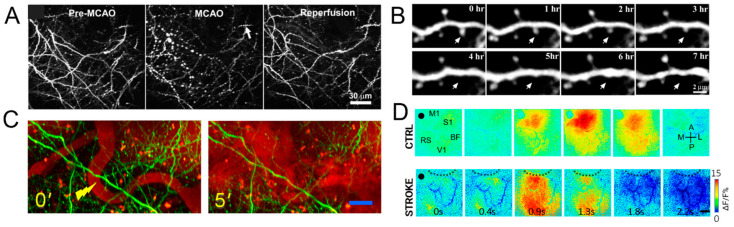
(**A**) Two-photon imaging of local changes in the dendritic structure before, during, and after MCAO. In the left panel, intact dendritic structures were observed; in the middle panel, extensive dendritic blebbing was observed; in the right panel, a significant recovery of dendritic structures after reperfusion was observed. Modified by Li and Murphy 2008, Copyright (2008) Society for Neuroscience. (**B**) Time-lapse imaging of apical dendrites showed the retraction of a dendritic spine. Modified by Brown et al., 2007, Copyright (2007) Society for Neuroscience. (**C**) Time-lapse images of maximum intensity z-projections (from 20 to 60 μm) before (left) and after (right) the laser-induced ischemic hemorrhage. The figures shown in green are the GFP-labeled neurons in a GFP-M mouse, and in red are the vascular networks labeled with Texas-red dextran dye. The tip of the yellow lightning symbol represents the laser irradiation point. The first image was acquired just before the laser irradiation. Scale bar, 20 μm. Modified from Allegra Mascaro et al., 2010. (**D**) Image sequences of cortical activation as assessed by calcium imaging during pulling of the handle by the contralateral forelimb of CTRL (top), STROKE (bottom) Thy1-GCaMP6f mice in the M-Platform. A small area located in the motor-sensory region reproducibly lit up in CTRL mice, while a large area covering most of the cortical surface of the injured hemisphere was activated in STROKE mice 1 month after stroke. A–P, anterior posterior, M-L, medio-lateral, M1, primary motor area, V1, primary visual area, S1, primary sensory area, Rs, Retro splenial area, BF, barrel field. The black dashed lines define the lesion borders. The black dot indicates bregma. Scale bar 1 mm. Modified from Allegra Mascaro et al., 2019.

Assessing the extension and progression of the CE and HT may not be sufficient to understand the reasons for the discrepancies in the clinical cases reported above. As described before, the entire NVU is involved in the degradation process triggered by cerebral ischemia. In turn, this cascade of events affects brain organization at all levels, from single synapses to neuronal networks to whole-brain activity. More in-depth understanding of the ischemic progression that leads to neuronal survival or massive degeneration in the penumbral tissue with cellular and subcellular detail is necessary. Preclinical research, though it is still not always able to reproduce the complexity and the variety of human clinical cases, presents the great advantage of dissecting neuronal structure and function over multi-scale. In the last decades, the development of 2PFM [99], coupled with the introduction of transgenic mice expressing genetically encoded fluorescent indicators in cortical neurons [100], has enabled investigators to visualize longitudinal changes in the structure of dendritic spines in vivo. In particular, many studies have focused on structural and functional plasticity as targets of both acute and chronic ischemia [101,102,103,104]. These studies indicated the loss of spines and rapid swelling and beading of dendritic structure within minutes of global ischemia coincident with a wave of ischemic depolarization [103]. Many studies [82,101,102] focused their attention on spines’ turnover and dendritic orientation in the peri-infarct cortex. In particular, dendritic structures can be profoundly altered by MCAO [103], whereas reperfusion can lead to recovery of structure similar to pre-stroke levels. In another work, Murphy and collaborators [105] demonstrated that the capability of dendritic arbors to recover within the penumbra was still maintained after 60 min of sustained ischemia (Figure 6A). By exploiting in vivo 2PFM and laser speckle contrast imaging, they correlated dendritic blebbing with the fluctuation of blood flow, showing that the recovery of the dendritic structure following reperfusion is restricted to a relatively small penumbra region. Brown and colleagues [106] took advantage of 2PFM to monitor real-time changes in dendritic and vascular structure in a mouse model of photothrombotic stroke (Figure 6B). In parallel, other studies investigated blood flow before and after multiphoton nano surgery of single blood vessels in living animals [107] (Figure 6C). 2P real-time imaging of blood flow through the blood vessels in the region of the cortex surrounding the vascular lesion permits the characterization of the dynamics of the degenerative event [107]. Since the reorganization of surviving cortical areas is involved in post-stroke recovery, in the last decades, neuroscience pointed their attention to functional in vivo studies too. Harrison and colleagues [108] investigated functional rearrangement between cortical regions in a mouse model of photothrombotic infarct targeted in the motor cortex. In this longitudinal study, they observed, through a combination of sensory-motor stimulations and intrinsic optical signal imaging, which spared regions of the cortex surrounding the stroke core were able to assume functions from stroke affected areas. Thereafter, Lim and collaborators, by taking advantage of voltage sensitive dye and optogenetic cortical stimulation, investigated neural rearrangement of cortical networks in the mouse brain cortex [109]. This relatively noninvasive approach allows recording neuronal activity triggered by optogenetic stimulation with high temporal resolution and large spatial resolution. This work provided evidence of the global depression of cortical activity characterizing the early stages after stroke. Moreover, they observed at a later time point (8 weeks after stroke) that the global depression gradually resolved, though the overall strength of the network remained reduced. Recently, Allegra Mascaro et al. [82] performed a multi-scale study investigating structural and functional plasticity in parallel, in a mouse model of post-stroke rehabilitation. More in detail, they observed in the peri-infarct cortex an increase in spines’ surviving fraction and a preferential orientation of dendrites towards the stroke core. Moreover, by investigating cortical activity during the execution of a motor task, they observed a widespread activation in chronic conditions (Figure 6D).

In line with these preclinical studies, a similar system-level measure of functional connectivity in humans through fMRI observed a consistent decrease in brain modularity indicating a reduction in integration within functional areas and segregation between brain systems during a subacute phase after store [110].

## 4. Conclusions

The limited knowledge on the molecular, cellular, and network level that can be offered by common clinical practice hinders the understanding of the mechanism underlying a specific clinical outcome. Within this review, we discussed the potentials and limits of preclinical research to answer clinical questions raised by the reported exemplary cases. Among others, the two major questions that this review tried to address were: (1) why does MT not improve stroke outcome in all patients, despite full recanalization? (2) how can stroke reperfusion treatments be further improved? Preclinical researchers are thrilled to bring their contribution to stroke care in a way that works alongside stroke clinicians and helps get the right patient to the right treatment (precision medicine), but there is still plenty of work to be done.

The last two decades have witnessed a remarkable increase in the number, breadth, and depth of preclinical research studies on acute ischemic stroke [111], but most of them carry some key mismatches between clinical practice and preclinical models:

- Stroke is most prevalent in elderly men and women, whereas preclinical models mostly test young animals.
- Stroke is more devastating in patients with multiple comorbidities not often captured by preclinical models.

However, exploratory research aimed at investigating potential new therapeutic targets or theoretical understanding of pathophysiological mechanisms does not necessarily need to perform experiments on an extensive range of age and comorbid models. Furthermore, since the incidence of stroke in young adults has increased in the last decades [112,113], preclinical research with young animals represents a fundamental way for understanding the underlying pathophysiological mechanisms in this subgroup of patients. Finally, the capability to investigate, at multiple-scale with different approaches, the ischemic progression from the onset up to the chronic phase after the insult allows the deep understanding of the post-stroke transformation, disentangled from other factors.

Unlike clinics, where HT and CE are used to define the prognosis of stroke patients, preclinical studies usually characterize the severity of the insult with behavioral tests in vivo or through the evaluation of the lesion volume ex-vivo. Nevertheless, since cerebral hemorrhage and edema are the most frequent clinical complication in the acute phase after an ischemic stroke, we illustrated that preclinical research had developed a multifaceted array of techniques to investigate these pathological processes. Another emerging topic that is catching the attention of preclinical research is the understanding of the role of cortical depolarization waves in the acute phase after stroke. Many studies [114,115,116,117,118], both in animals and humans, suggest that spreading depression-like depolarizations play a crucial role in the tissue damage process. Indeed, in the early stages after focal cortical ischemia, spreading depolarization waves propagate from the rim of the stroke core to the surrounding intact tissue [119,120]. Previous investigations [121] showed that in the injured brain, the succession of spreading depolarizing waves induces a series of intracellular alterations (i.e., collapses ionic gradients, activation of NMDA receptors and gap junctions), triggering a massive calcium influx that in energy-compromised neurons promotes the cell death cascade. Moreover, other studies highlighted the crucial role of the interaction between cortical spreading depression waves and the brain’s vasculature since in pathological conditions, they induce severe vasoconstriction and spreading ischemia [122,123]. Balbi and collaborators revealed the propagation of depolarizing waves by inducing a photothrombotic stroke in awake mice without the interference of anesthesia throughout the entire cortex [71]. Moreover, a recent work [124], by simultaneously investigating the neurovascular coupling during and following photothrombosis, identified a determining role of cortical spreading depression waves in the secondary progression of tissue damage during and after acute brain injury, emphasizing their potential therapeutic target. Though up to now the finest cellular and molecular pathophysiological mechanisms of ischemic progression are still largely unknown, future preclinical research should flank the characterization of hemorrhage and edema to neurovascular investigation in order to understand the mechanisms underlying the FR and define better therapeutic paradigms.

To this aim, neuroscientists are making a great effort in order to optimize animal models of stroke with reperfusion to investigate HT and CE in parallel with neuronal functionality and structural plasticity of synaptic contact, better resembling clinic progression observed in humans.

In conclusion, the ongoing technological development of cutting-edge investigation approaches will offer the capability to realize a more specific and detailed investigation of the pathophysiological mechanisms underlying ischemic progression. Undoubtedly, the bi-directional collaborative approach between preclinical and clinical researchers represents a propulsive thrust to improve stroke treatments.

## Data Availability

The data that support the findings of this study are available from the corresponding author upon reasonable request.
